# Reliability and measurement error of anterior maximum voluntary bite force in children with juvenile idiopathic arthritis and healthy children

**DOI:** 10.1371/journal.pone.0280763

**Published:** 2023-01-20

**Authors:** Willemijn F. C. de Sonnaville, Michel H. Steenks, Nicolaas P. A. Zuithoff, Nico M. Wulffraat, Antoine J. W. P. Rosenberg, Caroline M. Speksnijder

**Affiliations:** 1 Department of Oral and Maxillofacial Surgery and Special Dental Care, University Medical Center Utrecht, Utrecht University, Utrecht, The Netherlands; 2 Julius Center for Health Sciences and Primary Care, University Medical Center Utrecht, Utrecht, The Netherlands; 3 Department of Pediatric Rheumatology and Immunology, Wilhelmina Children’s Hospital, University Medical Center Utrecht, Utrecht University, Utrecht, The Netherlands; University of Catanzaro, ITALY

## Abstract

In children with juvenile idiopathic arthritis (JIA) the temporomandibular joint (TMJ) can be involved. As a consequence, the oral function can be impaired due to joint and/or muscle involvement of the masticatory system with a negative influence on the maximum bite force. The aim of this cross-sectional study was to establish the reliability of AMVBF in children with JIA and healthy children. Children with JIA and healthy children conducted three attempts of AMVBF. The reliability of AMVBF measurement was determined by the intra-class correlation coefficient (ICC) by age, standard error of measurement (SEM), smallest detectable change (SDC), and limits of agreement (LoA). A total of 298 children with JIA and 168 healthy children were examined. The AMVBF measurements showed an good to excellent reliability in children with JIA based on the ICCs corrected for age (0.782–0.979). In healthy children, the reliability was moderate to excellent (0.546–0.999). The SDC in our study indicated that values above 11.4N might be a clinical relevant change over time in children with JIA. The LoA showed a wide spread of variability in both children with JIA (-72.6–44.4N) and healthy children (-79.9–72.8N). The Bland-Altman plots indicated that the differences between the test and retest increased in value proportionally to the biteforce value.

## Introduction

The voluntary bite force is mentioned as an indicator for the functional state of the masticatory system [1–3]. It has been studied in a variety of disciplines, such as temporomandibular pain and disorders, and in specific pathologies of the masticatory system [4, 5]. Furthermore, various devices and methods have been described to determine bite force, such as the type of transducer, strain-gauge, piezoelectric and pressure transducers, as well as the position of the transducer in between the teeth of the upper and lower dental arch (front teeth bite force or molar bite force) [4]. As a result, the bite force can vary per device, method and study population [6]. In children with juvenile idiopathic arthritis (JIA) the temporomandibular joint (TMJ) can be affected. JIA is diagnosed when arthritis is characterized by inflammation of one or more joints and is present for at least six weeks with an onset before the age of 16 [7]. Epidemiological data of TMJ involvement prevalence vary widely (30–96%), depending on the definition of TMJ involvement, diagnostic methods and characteristics of study participants [8–11]. As a consequence of this TMJ involvement, the oral function can be impaired due to joint and/or muscle involvement of the masticatory system with a negative influence on the maximum bite force. In previous studies, the anterior maximum bite force in children with JIA was 24.0 Newtons (N) lower compared to healthy children, and even 42N lower in children with JIA and TMJ involvement [10, 12]. To appraise such a reduction, it is necessary to gain more insight in the reliability and smallest detectable change (SDC) of the maximum bite force in children with JIA and in healthy children. Monitoring the TMJ in a longitudinal evaluation is advised as an optimal management in patients with JIA [13]. Clinicians may interpret changes over time regarding bite force easier than absolute values. The interpretation of changes over time warrants knowledge regarding natural fluctuations in healthy children and in children with JIA: the SDC. The SDC is a concept to support the clinician in treatment decisions. The paediatric rheumatologist, following up on the TMJ status in children with JIA, is alerted whether the change over time is within the SDC or exceeds the SDC of AMVBF since this may indicate a clinically relevant change. For these reasons, quantifying the SDCs, intra-class correlation (ICC) and limits of agreement (LoAs) for bite force in children with JIA is a clinically relevant procedure. Therefore, the aim of this study was to establish the reliability of anterior maximum voluntary bite force (AMVBF) in children with JIA and in healthy children.

## Materials and methods

### Children with JIA

The AMVBF measurements in children with JIA were performed between January 2018 and February 2020 at the outpatient clinic of the Department of Pediatric Immunology and Rheumatology in collaboration with the Department of Oral and Maxillofacial Surgery and Special Dental Care of the University Medical Center (UMC) Utrecht, The Netherlands. The AMVBF measurements were carried out immediately after a regular consultation with the paediatric rheumatologist.

The inclusion criteria for participation were children with: 1) JIA as classified using the International League of Associations for Rheumatology criteria and 2) an age between 6 and 18 years old. Exclusion criteria were: 1) a history of mandibular trauma; 2) previous TMJ treatment, such as physical therapy, occlusal splints, intra-articular injections or maxillofacial surgery; 3) incisal dental restoration or non-erupted incisors; 4) an additional orofacial condition not related with JIA (e.g. dental pain or a pre-existing jaw or temporomandibular disorder); and 5) children who were not able to complete at least two AMVBF attempts.

We extracted the following data from the electronic medical record for these children: JIA subtype, date of JIA diagnosis, medication, length, weight, gender, age, the presence of antinuclear antibody (ANA) or rheumatoid factor (RF), and the clinical Juvenile Arthritis Disease Activity Score (cJADAS) [14].

### Healthy children

Healthy children were recruited and measured at primary schools in Utrecht and a high school in Tilburg, The Netherlands, between February 2018 and April 2019. The inclusion criteria for healthy children were: 1) age between 6 and 18 years old. The exclusion for healthy children were: 1) a history of mandibular trauma; 2) previous TMJ treatment, such as physical therapy, occlusal splints, intra-articular injections or maxillofacial surgery; 3) incisal dental restoration or non-erupted incisors; 4) an additional orofacial condition not related with JIA (e.g. dental pain or a pre-existing jaw or temporomandibular disorder); 5) children who were not able to complete at least two AMVBF attempts; and 6) a score of at least 2 on the TMJ screening protocol (n = 12) [15].

The study was carried out in accordance with The Code of Ethics of the World Medical Association. The study protocol, with study ID NL.METC-17-528/C, was approved by the Ethics Committee of the UMC Utrecht. All participants and their parents and/or guardians received written information and provided oral and signed informed consent. Data collection was performed using the Good Clinical Practice compliant Electronic Data Capture system Research Online. The proprietary Electronic Data Capture system is owned by the Julius Center at the UMC Utrecht.

### Anterior maximum voluntary bite force

The AMVBF was measured both in children with JIA and healthy children using a bite force transducer, based on the bite force transducer from the Amsterdam University Medical Center and further developed by the University Medical Center Utrecht [16]. The bite force gauge is a handheld device with a load cell to measure AMVBF, with a range between 0 and 490N in a linear fashion. The device consists of a strain gauge mounted on a mouthpiece of 10x15 mm and a thickness of 12 mm. Plastic foil was applied around the mouthpiece for each child to guarantee hygiene. The mouthpiece was placed between the upper and lower central incisors. The bite force measurement consisted of clenching, as hard as possible for ten seconds, at maximum. In one session, three attempts, AMVBF_1_, AMVBF_2_ and AMVBF_3_, were documented and expressed in N. In between the three attempts, the children indicated when they were ready for the next attempt. All participants were instructed and encouraged in a similar way through a taped voice recording.

### Statistical analysis

The characteristics of the children are presented as numbers and percentages, or as means and standard deviations (SD). For the analyses of demographic and clinical data, the unpaired sample t-test was used for continuous data, and the chi-squared test was used for dichotomous or ordered categorical outcomes. Data were graphically evaluated on normal distribution.

The test-retest reliability was checked for the three measurements and depicted as AMVBF_1-2_, AMVBF_2-3_ and AMVBF_1-3_, and calculated by the two-way random, absolute agreement, single measurement intraclass correlation coefficient (ICC_2,1_) and the associated 95% confidence intervals (CI). The ICC was calculated as $\frac{MS_{R}-MS_{E}}{MS_{R}+\left(k-1\right)MS_{E}+\frac{k}{n}\left(MS_{C}-MS_{E}\right)}$ in which MS_R_ = mean square of rows, MS_E_ = mean square of error, k = number of measurements and n = number of subjects. The ICC explains the consistency of the measurements, and the cut-off points for the ICC were chosen as poor (<0.50), moderate (0.50–0.75), good (0.75–0.90) and excellent (>0.90) [17, 18]. In addition the ICC was corrected for age, as it can substantially overestimate the reliability [19]. The standard error of measurements (SEM) was calculated as SEM = SD* √ (1−ICC), with SD defined as the difference between the two AMVBFs. The SEM explains how much the values of the measurements of the test and retest differ from each other. The SEM percent change was calculated as SEM% = (SEM/$\bar{X}$) ×100, in which $\bar{X}$ the mean of all measurements of test and retest.

The SDC was calculated as SDC = 1.96×√2×SEM [20]. The SDC is the smallest statistically significant change of AMVBF that the bite force measurement can detect in individuals. The SDC percent change was calculated as SDC% = ($\text{SDC}/\bar{X}*100$, in which $\bar{X}$ is the mean of all measurements of test and retest.

In order to check for proportional bias, variability and agreement, Bland-Altman plots were constructed by plotting the test-retest difference versus the mean value of the test and retest [21]. Agreement between test and retest was summarized using the mean difference and SD of the difference, and the 95% limits of agreement (LoA) were calculated as LoA = Mean±1.96×SD [21]. The LoA estimated the interval of the difference between the test and the retest. A p-value of less than 0.05 was accepted as significant. Statistical analyses were performed using SPSS 25 (IBM SPSS Statistics for Windows, Version 25.0. Armonk, NY: IBM Corp).

## Results

A total of 290 children with JIA and 168 healthy children were examined in this study. Of all measurements, in one healthy child AMVBF_1,2,3_ was missing due to an open bite. In children with JIA, missing values were found of AMVBF_1,2,3_ in eight individuals (technical problems (n = 2), mobility of the front teeth due mixed dentition (n = 2), pain of the front teeth (n = 1), misalignment of the front teeth (n = 1), no coordination to bite properly on the bite force transducer (n = 1), unknown (n = 1)). These missing values were excluded from the analyses.

### Children with JIA

Children with JIA had a mean age of 12.8 years (SD 3.4), and 197 (67.9%) of them were girls (Table 1). AMVBF_2_ values were missing in two children with JIA due pain in the front teeth, while AMVBF_3_ was missing in 11 children with JIA (due to not properly biting on the bite force transducer), pain in the front teeth (n = 4) and unknown reasons (n = 6)). In total, 288 measurements of AMVBF_1-2_, 278 of AMVBF_2-3_ and 279 of AMVBF_1-3_ were analysed. The disease characteristics of the children with JIA are presented in Table 1. The mean AMVBF_1_ for children with JIA was 114.9 N (SD 57.5), AMVBF_2_ 118.2 N (SD 59.1) and AMVBF_3_ 133.5 N (SD 62.4) (Table 1).

**Table 1:** Demographics and AMVBF of children with JIA and healthy controls.

	JIA (n = 290)	Healthy children (n = 168)	*P*-value
Gender (n, %)			<0.001^a^
Male	93 (32.1)	87 (51.8)	
Female	197 (67.9)	81 (48.2)	
Mean age (years; mean, SD)	12.8 (3.4)	11.4 (3.5)	<0.001^b^
Mean weight (kg; mean, SD)	50.9 (17.5)	46.7 (17.2)	0.014^b^
Mean height (cm; mean, SD)	157.4 (17.9)	152.8 (20.9)	0.018^b^
Orthodontic treatment (n, %)	47 (16.2)	17 (10.1)	0.070^a^
Medication use (n, %)	220 (75.9)	14 (8.3)	<0.001^a^
Clinical remission off medication	70 (24.1)		
JIA subtype (n, %)			
Systemic	29 (10.0)		
Oligoarticular, persistent	110 (37.9)		
Oligoarticular, extended	31 (10.7)		
Polyarticular, RF-	61 (21.0)		
Polyarticular, RF+	15 (5.2)		
Enthesitis-related	17 (5.9)		
Psoriatic arthritis	15 (5.2)		
Undifferentiated	12 (4.1)		
Laboratory studies (n, %)			
Positive ANA	91 (31.4)		
Positive RF	14 (4.8)		
Positive HLA-B27	21 (7.2)		
Mean disease duration (months; mean, SD)	62.4 (51.8)		
cJADAS (n, %)			
0–2 (low)	182 (62.8)		
3–7 (moderate)	60 (20.7)		
≥8 (high)	42 (14.5)		
Missing	6 (2.1)		
**Medication use (n, %)**			
NSAIDs	87 (30.0)		
Corticosteroids	14 (4.8)		
DMARDs	134 (46.2)		
Biologicals	86 (29.7)		
No medication	70 (24.1)		
DMARDS (n, %)			
Methotrexate	115 (39.7)		
Leflunomide	12 (4.1)		
Azathioprine	2 (0.7)		
Sulfasalazine	2 (0.7)		
Other	3 (1.0)		
No DMARDs	156 (53.8)		
Biologicals (n, %)			
Adalimumab	40 (13.8)		
Etanercept	23 (7.9)		
Tocilizumab	6 (2.1)		
Canakinumab	5 (1.7)		
Golimumab	5 (1.7)		
Abatacept	1 (0.3)		
Anakinra	2 (0.7)		
Infliximab	1 (0.3)		
Other	3 (1.0)		
No biologicals	204 (70.3)		
AMVBF_1_ (mean, SD)	114.9 (57.5)	137.0 (63.3)	<0.001^b^
AMVBF_2_ (mean, SD)	118.2 (59.1)	133.6 (62.7)	0.010^b^
AMVBF_3_ (mean, SD)	133.5 (62.4)	142.4 (63.7)	0.148^b^

^a^ chi-squared test;

^b^ independent sample t-test.

ANA: antinuclear antibody; AMVBF: anterior voluntary bite force; cJADAS: Clinical Juvenile Arthritis Disease Activity Score; DMARDs: disease-modifying anti-rheumatic drugs; HLA-B27: human leukocyte antigen B27; JIA: juvenile idiopathic arthritis; NSAIDs: non-steroidal anti-inflammatory drugs; RF: rheumatoid factor; SD: standard deviation; TMJ: temporomandibular joint.

**Fig 1:**
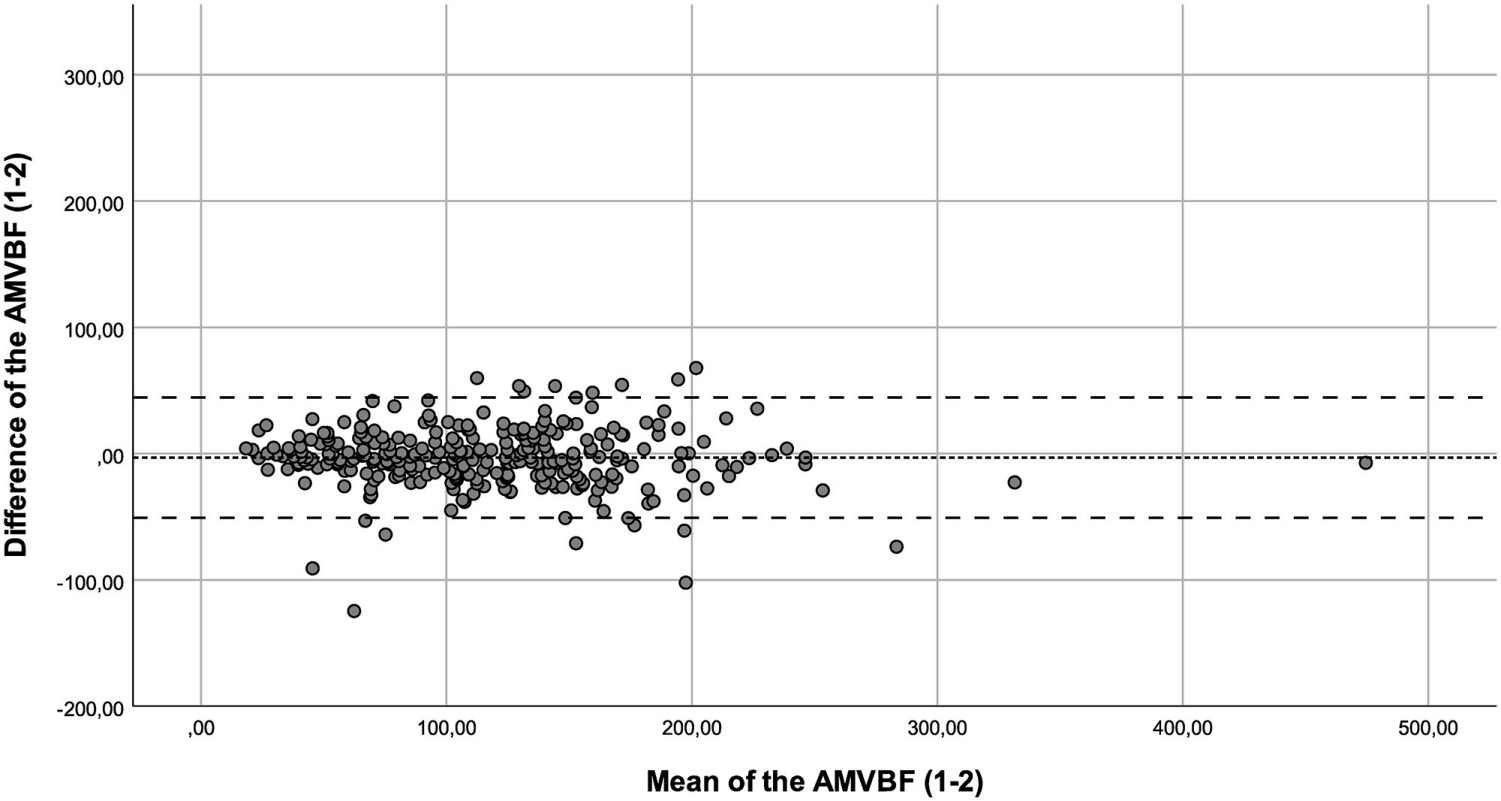
Bland-Altman plot for the difference between test and retest (test 1–2) of the anterior maximum voluntary bite force (AMVBF) for patients with JIA. The dashed line represents the mean difference between test and retest, and the striped lines represent the 95% limits of agreement.

**Fig 2:**
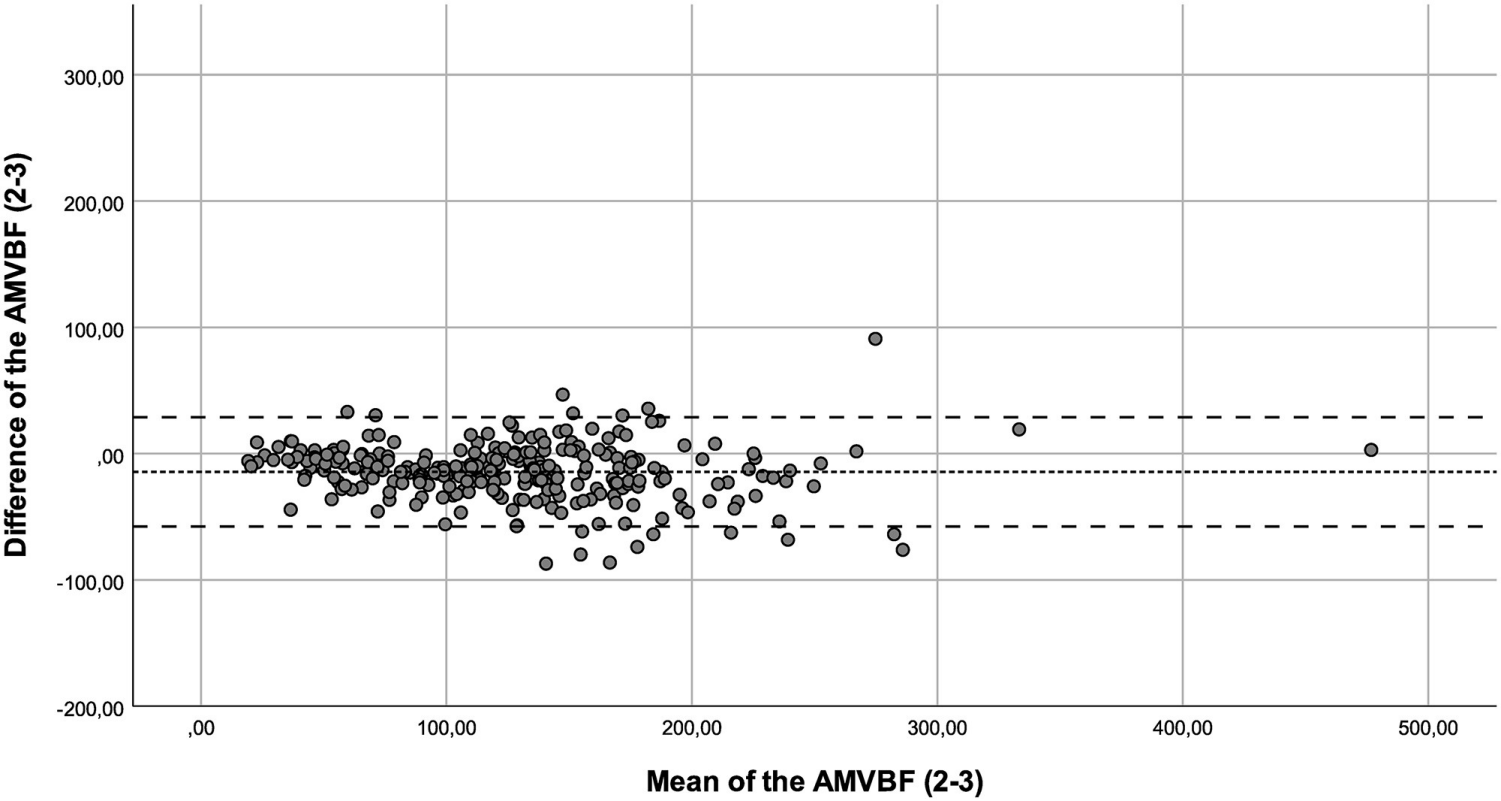
Bland-Altman plot for the difference between test and retest (test 2–3) of the anterior maximum voluntary bite force (AMVBF) for patients with JIA. The dashed line represents the mean difference between test and retest, and the striped lines represent the 95% limits of agreement.

**Fig 3:**
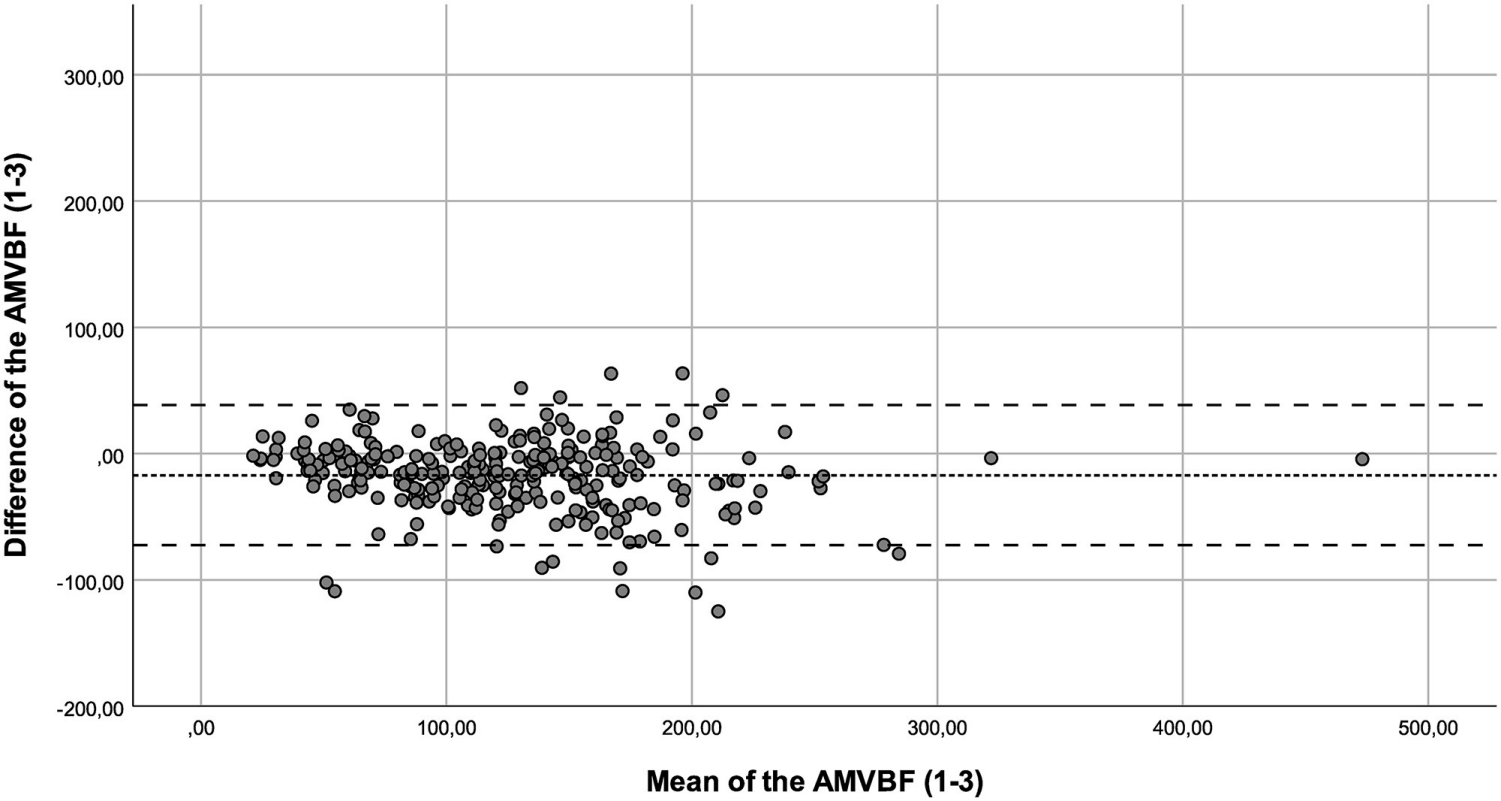
Bland-Altman plot for the difference between test and retest (test 1–3) of the anterior maximum voluntary bite force (AMVBF) for patients with JIA. The dashed line represents the mean difference between test and retest, and the striped lines represent the 95% limits of agreement.

The ICC showed a good correlation between the of AMVBF_1-3_ (ICC = 0.855), and an excellent ICC for AMVBF_1-2_ (ICC = 0.913) and AMVBF_2-3_ (ICC = 0.909; Table 2). The ICCs corrected for age of AMVBF_1,2,3_ varied between 0.782 and 0.979 and showed a good to excellent correlation (Table 3). The SEM for children with JIA was 2.1 N, 2.0 N and 4.1 N with a SEM% of 1.8%, 1.6%, and 3.3% for AMVBF_1-2_, AMVBF_2-3_ and AMVBF_1-3_, respectively. The SDC was 5.9 N, 5.6 N and 11.4 N, with an SDC% of 5.0%, 4.4% and 9.1% for AMVBF_1-2_, AMVBF_2-3_ and AMVBF_1-3_. The LoA varied between -72.58 and 44.4 (LoA; AMVBF_1-2_ = -50.87–44.39, AMVBF_2-3_ = -57.77–28.87 and AMVBF_1-3_ = -72.58–38.36; Figs 1–3; Table 2).

**Table 2:** Reliability of the anterior voluntary maximum bite force (AMVBF) for children with JIA and healthy children.

	JIA			Healthy children		
	AMVBF_1-2_	AMVBF_2-3_	AMVBF_1-3_	AMVBF_1-2_	AMVBF_2-3_	AMVBF_1-3_
	(n = 288)	(n = 278)	(n = 279)	(n = 168)	(n = 168)	(n = 168)
Difference test-retest mean (SD)	-3.24 (24.3)	-14.45 (22.1)	-17.11 (28.3)	3.39 (35.4)	-8.86 (33.9)	-5.46 (38.0)
ICC	0.913	0.909	0.855	0.842	0.849	0.819
95% CI	0.891–0.930	0.769–0.953	0.690–0.919	0.792–0.881	0.793–0.889	0.762–0.863
SEM	2.11	2.01	4.10	5.59	5.12	6.88
SEM%	1.81%	1.58%	3.27%	4.14%	3.71%	4.92%
SDC	5.85	5.57	11.37	15.50	14.19	19.06
SDC%	5.03%	4.39%	9.06%	11.46%	10.28%	13.65%
95% LoA	-50.87–44.39	-57.77–28.87	-72.58–38.36	-65.99–72.77	-75.30–57.58	-79.94–69.02

Abbreviations: AMVBF: anterior maximum voluntary bite force; CI: confidence interval; ICC: intra-class correlation coefficient; LoA: limits of agreement; SDC: smallest detectable change; SDC%: SDC/mean of all measurements of test and retest; SEM: standard error of measurement; SEM%: SEM/mean of all measurements of test and retest.

**Table 3:** Intra class-correlation corrected for age of the anterior voluntary maximum bite force (AMVBF) for children with JIA and healthy children.

	JIA		Healthy children	
Age	N	ICC	N	ICC
6	17	0.964	7	0.821
7	10	0.979	25	0.738
8	16	0.852	13	0.651
9	19	0.833	19	0.868
10	16	0.847	12	0.812
11	18	0.865	11	0.843
12	24	0.965	9	0.899
13	31	0.919	11	0.894
14	28	0.855	20	0.932
15	34	0.933	16	0.546
16	38	0.820	11	0.880
17	23	0.956	12	0.589
18	16	0.782	2	0.999

AMVBF: anterior maximum voluntary bite force; ICC: intra-class correlation coefficient.

The ICC was calculated by using data for AMVBF_1,2,3_.

### Healthy children

In the group of healthy children, the mean age was 11.4 years old (SD 3.5) and 81 (48.2%) were girls (Table 1). A total of 168 measurements were analysed for AMVBF_1-2_, AMVBF_2-3_ and AMVBF_1-3_. The mean AMVBF for healthy children for attempt 1 was 137.0 N (SD 63.3), attempt 2 133.6 N (SD 62.7), and attempt 3 142.4 N (SD 63.7) (Table 1).

**Fig 4:**
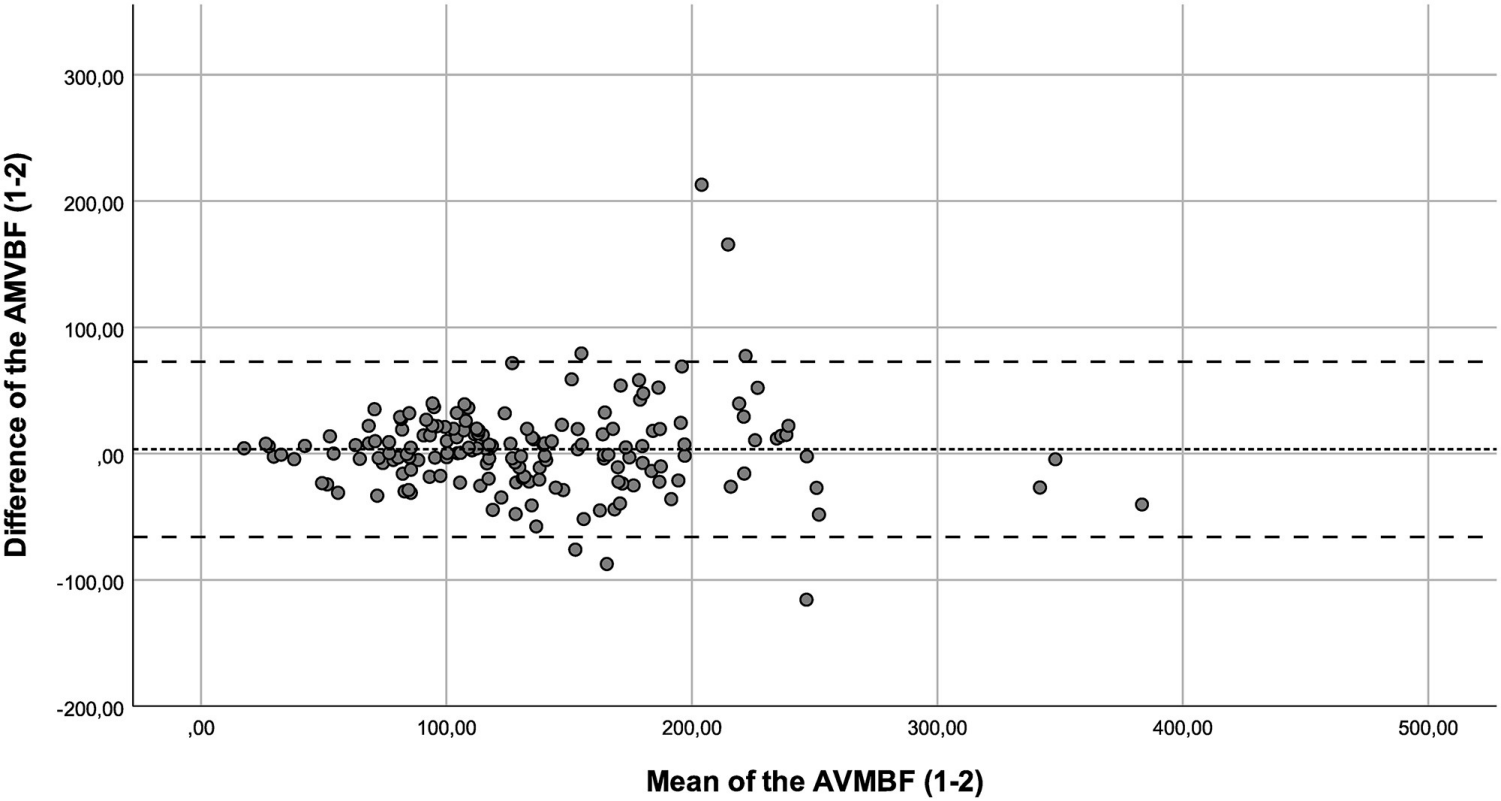
Bland-Altman plot for the difference between test and retest (test 1–2) of the anterior maximum voluntary bite force (AMVBF) for healthy children. The dashed line represents the mean difference between test and retest, and the striped lines represent the 95% limits of agreement.

**Fig 5:**
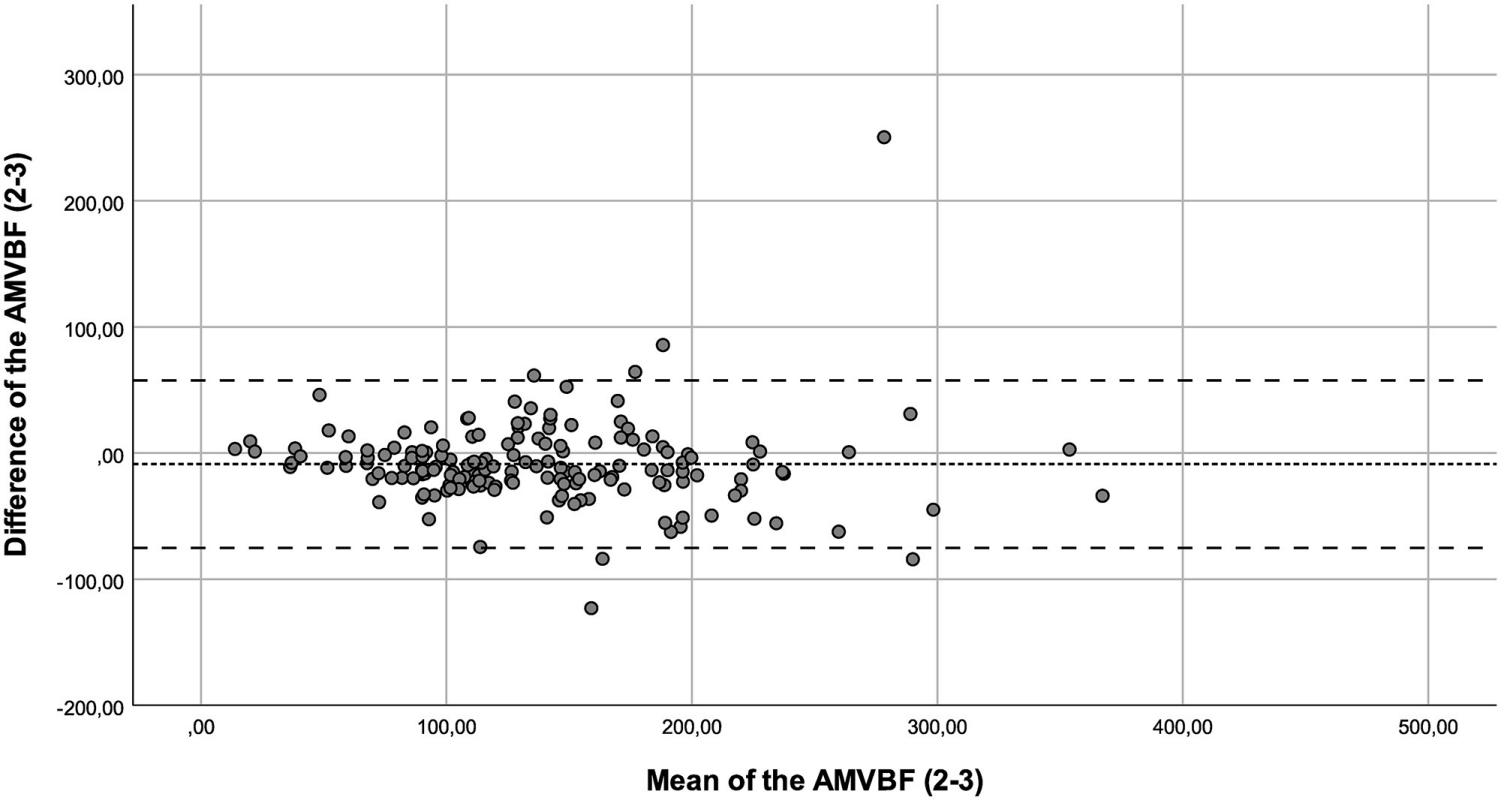
Bland-Altman plot for the difference between test and retest (test 2–3) of the anterior maximum voluntary bite force (AMVBF) for healthy children. The dashed line represents the mean difference between test and retest, and the striped lines represent the 95% limits of agreement.

**Fig 6:**
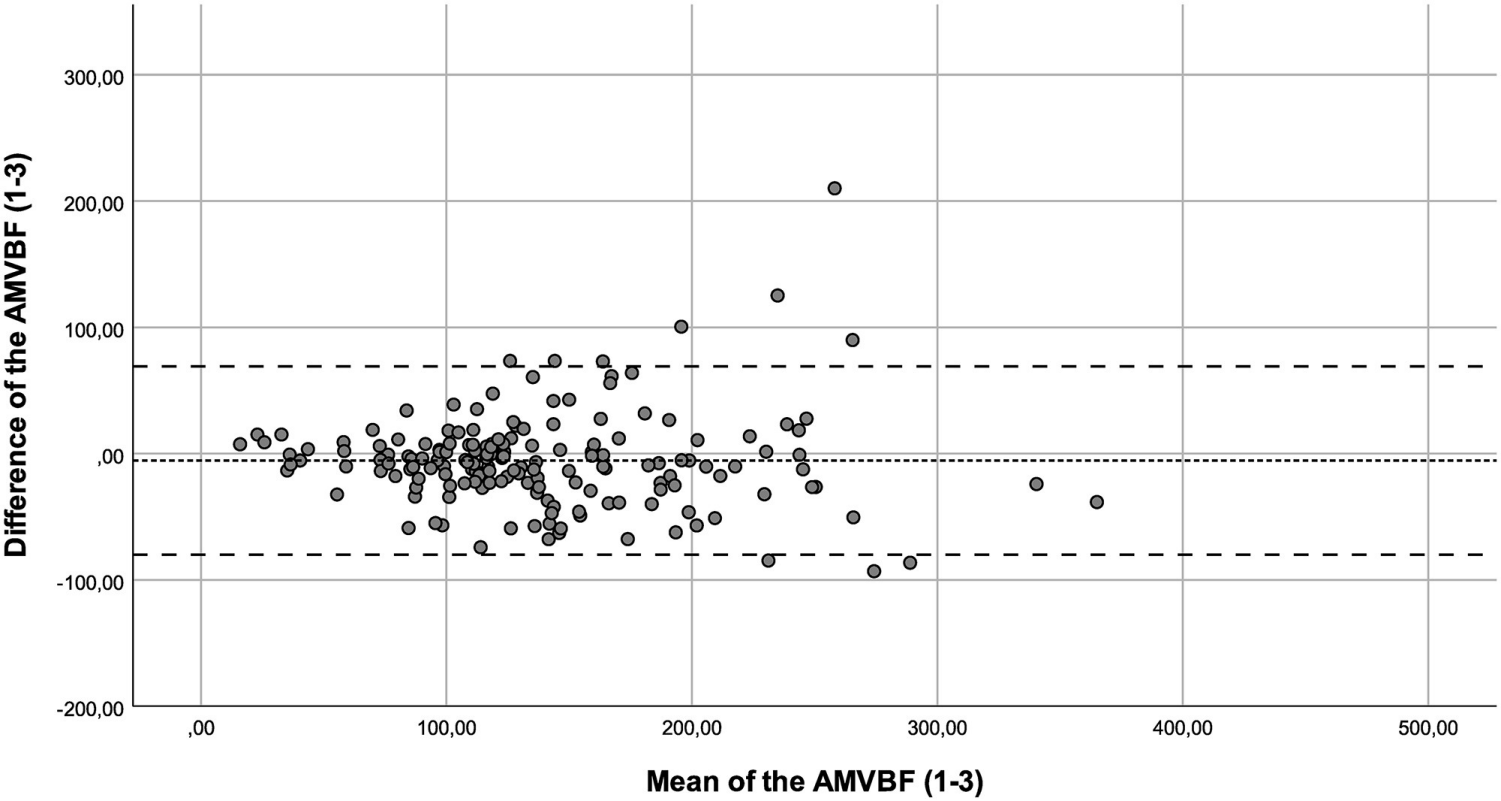
Bland-Altman plot for the difference between test and retest (test 1–3) of the anterior maximum voluntary bite force (AMVBF) for healthy children. The dashed line represents the mean difference between test and retest, and the striped lines represent the 95% limits of agreement.

The ICC of AMVBF_1-2_ (ICC = 0.842), AMVBF_2-3_ (ICC = 0.849), AMVBF_1-3_ (ICC = 0.819) showed a good correlation. The ICCs corrected for age of AMVBF_1,2,3_ varied between 0.546 and 0.999 and showed a moderate to excellent correlation (Table 3). The SEM was 5.6, 5.1 and 6.9 with a SEM% of 4.1%, 3.7% and 4.9% for AMVBF_1-2_, AMVBF_2-3_ and AMVBF_1-3_, respectively. The SDC for healthy children was 15.5 N, 14.2 N and 19.1 N, with an SDC% of 11.5%, 10.3% and 13.7% for AMVBF_1-2_, AMVBF_2-3_ and AMVBF_1-3_. The LoA varied between -79.94 and 72.77 (LoA; AMVBF_1-2_ = -65.99–72.77, AMVBF_2-3_ = -75.30–57.58 and AMVBF_1-3_ = —79.94–69.02; Figs 4–6, Table 2).

Outliers were identified through the Bland-Altman plots. We checked these outliers for procedural errors. No explanation was found for the outliers, and therefore we interpreted these as normal fluctuations.

## Discussion

The reliability of AMVBF in children with JIA and in healthy children was evaluated by determining the ICC, SEM, SDC, LoA and Bland-Altman plots. The established ICCs showed at least good reliability in children with JIA. The ICCs corrected for age showed in children with JIA good to excellent ICCs and in healthy children moderate to excellent ICCs. While the ICC is a relative measure of reliability, the SEM is an absolute index for reliability. The SEM in children with JIA varied between 2.0 N and 4.1 N, and in healthy children between 5.1 N and 6.9 N. This is relatively small compared to the mean of the measured AMVBFs (114.9–142.4 N; Table 1). Moreover, the SEM values are below the SDC, varying between 5.6 N and 19.1 N. A difference of 11.4 N (9.1%) can at least be detected in children with JIA, and 19.1 N (13.7%) in healthy children as a true difference instead of the result of measurement uncertainty. The LoA showed a wide spread of variability in both children with JIA and healthy children, with the highest ranges in AMVBF_1-3_.

The ICC showed good to excellent reliability in both children with JIA and healthy children. However, ICC shows the degree of association and not agreement [22]. In our data, we found a trend for increasing bite force levels during the attempts. This is also reflected in the mean AMVBF; AMVBF_3_ showed the highest value (Table 1). A possible explanation for this might be a learning effect, i.e. getting used to the bite force transducer. During data collection, we also noticed a competitive effect during the measurements: in the third attempt, children wanted to reach the highest possible bite force.

Additionally, we noticed in the Bland-Altman plots that the differences between the test and retest increased in value proportionally to the bite force value. This offers nuance regarding the calculated wide ranges of the LoA. In such cases, a transformation of the data on a log scale can be useful [22]. The log transformed data provide the spread of variability adjusted to the amount of AMVBF. The difficulty in interpreting such log transformed variables for clinical use is a main concern [23]. Therefore, we decided not to use transformation on a log scale. The LoA are probably too wide for small AMVBF values, and probably too narrow for large AMVBF values. As a result, lower AMVBF values seem to be more reliable than larger AMVBF values. This finding may be relevant, as the main clinical focus is on lower AMVBF values in children with JIA. Lower AMVBF values were found in children with JIA and TMJ involvement, while higher AMVBF values were found in children with JIA without TMJ involvement and in healthy children [10, 12].

Greater variability has been reported in older healthy children than in younger healthy children [19, 24]. Therefore, ICCs corrected for age are recommended. In our study, we found good to excellent ICCs in all ages in children with JIA, while in healthy children, the ICC was moderate in children at the ages of 8, 15 and 17 years. However, we did notice greater variability in larger bite force measurements. This may correspond with the increase in bite force with each year of age, and the proportional increase in SD (S1 Table).

Most studies have examined molar bite force, generating a higher bite force value than those from the interincisal region [19, 25, 26]. The recording technique influences the bite force level, but also its variability [19]. Therefore, it is difficult to compare different recording techniques with each other [1, 25]. Interincisal bite force was found to have the highest reliability in healthy children [19]. Another reason to choose the interincisal bite force measurement is the more symmetrical bite than bite force measurements with unilateral molar bites. Moreover, the bite force transducer in the molar region will lead to a larger gape than interincisal bites, which might be technically more difficult to apply. This can be cumbersome in some younger children and in children with a restricted mouth opening, for example children with JIA. However, interincisal measurements may have some disadvantages as well. The pressure exerted on the front teeth may result in an unusual sensation that could have influenced the bite force results. In our data, one child did not complete all bite force attempts due pain of the front teeth, and was therefore excluded from the analysis. In addition, two attempts of AMVBF_2_ and four attempts of AMVBF_3_ were missing due to pain in the front teeth.

In conclusion, AMVBF measurements showed good to excellent reliability in children with JIA based on the ICCs corrected for age. In healthy children, the reliability was moderate to excellent. The SDC in our study indicated that values above 11.4N in children with JIA might be a clinical relevant change over time. In further studies the founded SDC can be useful for longitudinal monitoring of the AMVBF in children with JIA, as this is suggest as an optimal management in patients with JIA [13]. Wide ranges of LoA were found in both children with JIA and in healthy children. However, we noticed in the Bland-Altman plots that the differences between the test and retest increased in value proportionally to the bite force value. Therefore, the LoA are probably too wide for small AMVBF values, and probably too narrow for large AMVBF values. Overall, low AMVBF values in children with JIA seemed to have the highest reliability in our study. This finding may be useful, as the main clinical focus is on lower AMVBF values in children with JIA.

## Supporting information

S1 Table Mean AMVBF and SD by age. Abbreviations: AMVBF: anterior maximum voluntary bite force; JIA: juvenile idiopathic arthritis; SD: standard deviation.
(DOCX)

## Abbreviations

AMVBF: Anterior maximum voluntary bite force
ANA: Antinuclear antibody
CI: Confidence interval
cJADAS: Clinical Juvenile Arthritis Disease Activity Score
DMARDS: Disease-modifying anti-rheumatic drugs
EDC: Electronic Data Capture
GCP: Good clinical practice
HLA-B27: Human leukocyte antigen B27
ICC: Intra-class correlation coefficient
ILAR: International League of Associations for Rheumatology
JIA: Juvenile idiopathic arthritis
k: Number of measurements
LoA: Limits of agreement
MSE: Mean square
MSR: Mean square of rows
N: Newtons
n: Number of subjects
NRS: Numeric rating scale
NSAIDS: Non-steroidal anti-inflammatory drugs
RF: Rheumatoid factor
SD: Standard deviation
SDC: Smallest detectable change
SEM: Standard error of measurement
SPSS: Statistical Package for the Social Sciences
TMJ: Temporomandibular joint
UMC: University medical center
