# Supplementary material for: Reliability and measurement error of anterior maximum voluntary bite force in children with juvenile idiopathic arthritis and healthy children
Source: PLoS One. 2023 Jan 20;18(1):e0280763. doi: 10.1371/journal.pone.0280763 (PMC9858014; doi:10.1371/journal.pone.0280763)
Supplement: S1 Table — Abbreviations: AMVBF: anterior maximum voluntary bite force; JIA: juvenile idiopathic arthritis; SD: standard deviation. (DOCX) [file pone.0280763.s001.docx]

**Table S1 -** Mean AMVBF and SD by age

|  | JIA | | | Healthy | | |
| --- | --- | --- | --- | --- | --- | --- |
| **Age (years)** | **AMVBF_1_ (mean, SD)** | **AMVBF_2_ (mean, SD)** | **AMVBF_3_ (mean, SD)** | **AMVBF_1_ (mean, SD)** | **AMVBF_2_ (mean, SD)** | **AMVBF_3_ (mean, SD)** |
| 6 | 68.9 (50.11) | 72.6 (50.8) | 83.3 (50.8) | 46.86 (21.5) | 52.1 (24.0) | 45.7 (25.3) |
| 7 | 62.8 (52.7) | 65.3 (61.4) | 71.4 (65.7) | 93.9 (54.8) | 86.3 (39.9) | 99.1 (47.1) |
| 8 | 80.1 (32.3) | 85.6 (34.1) | 99.7 (40.1) | 115.9 (42.5) | 109.2 (33.0) | 121.2 (32.7) |
| 9 | 104.5 (36.4) | 106.5 (41.1) | 124.8 (33.1) | 131.9 (40.1) | 138.4 (46.6) | 143.0 (48.7) |
| 10 | 122.9 (40.2) | 117.8 (39.8) | 128.0 (39.5) | 137.4 (54.0) | 152.5 (57.0) | 157.6 (49.9) |
| 11 | 105.5 (54.4) | 119.1 (50.7) | 130.6 (47.1) | 149.0 (42.2) | 159.7 (54.1) | 170.5 (61.0) |
| 12 | 122.8 (54.5) | 127.8 (57.5) | 141.1 (59.9) | 145.4 (47.2) | 135.1 (42.5) | 152.0 (51.6) |
| 13 | 96.4 (49.8) | 98.7 (49.6) | 108.0 (53.5) | 133.3 (31.3) | 140.1 (61.8) | 146.1 (54.7) |
| 14 | 123.4 (41.7) | 127.1 (47.7) | 150.8 (51.9) | 156.7 (71.2) | 148.1 (73.8) | 158.4 (77.5) |
| 15 | 127.4 (57.4) | 132.5 (59.8) | 149.4 (61.5) | 187.0 (61.0) | 163.3 (41.7) | 175.5 (56.0) |
| 16 | 133.9 (58.1) | 132.1 (57.3) | 143.5 (56.4) | 155.9 (64.1) | 144.4 (61.2) | 173.4 (76.0) |
| 17 | 150.4 (90.3) | 149.0 (94.1) | 180.0 (96.6) | 167.4 (73.1) | 159.6 (83.9) | 147.4 (43.2) |
| 18 | 126.4 (43.3) | 134.4 (53.3) | 150.7 (61.3) | 203.7 (176.2) | 223.6 (186.2) | 221.9 (184.6) |

Abbreviations: AMVBF: anterior maximum voluntary bite force; JIA: juvenile idiopathic arthritis; SD: standard deviation.
